# *Vibrio cholerae* Hemolysin Is Required for Lethality, Developmental Delay, and Intestinal Vacuolation in *Caenorhabditis elegans*

**DOI:** 10.1371/journal.pone.0011558

**Published:** 2010-07-13

**Authors:** Hediye Nese Cinar, Mahendra Kothary, Atin R. Datta, Ben D. Tall, Robert Sprando, Kivanc Bilecen, Fitnat Yildiz, Barbara McCardell

**Affiliations:** 1 Division of Virulence Assessment, Food and Drug Administration, Laurel, Maryland, United States of America; 2 Department of Environmental Toxicology, University of California Santa Cruz, Santa Cruz, California, United States of America; 3 Division of Toxicology, Food and Drug Administration, Laurel, Maryland, United States of America; National Institute on Aging, United States of America

## Abstract

**Background:**

Cholera toxin (CT) and toxin-co-regulated pili (TCP) are the major virulence factors of Vibrio cholerae O1 and O139 strains that contribute to the pathogenesis of disease during devastating cholera pandemics. However, CT and TCP negative *V. cholerae* strains are still able to cause severe diarrheal disease in humans through mechanisms that are not well understood.

**Methodology/Principal Findings:**

To determine the role of other virulence factors in *V. cholerae* pathogenesis, we used a CT and TCP independent infection model in the nematode *Caenorhabditis elegans* and identified the hemolysin A (*hlyA*) gene as a factor responsible for animal death and developmental delay. We demonstrated a correlation between the severity of infection in the nematode and the level of hemolytic activity in the *V. cholerae* biotypes. At the cellular level, *V. cholerae* infection induces formation of vacuoles in the intestinal cells in a *hlyA* dependent manner, consistent with the previous *in vitro* observations.

**Conclusions/Significance:**

Our data strongly suggest that HlyA is a virulence factor in *C. elegans* infection leading to lethality and developmental delay presumably through intestinal cytopathic changes.

## Introduction

Understanding the nature of the biological determinants that underlie severe illness has been a longstanding goal of *V. cholerae* research. Cholera toxin (CT) and toxin-coregulated pili (TCP) are the major virulence determinants of *V. cholerae* O1 and O139 strains. However, other *V. cholerae* sero groups and vaccine strains that lack CT and TCP are also capable of causing diarrheal illness [1], [2], [3], [4]. In addition to these two virulence factors, other toxins such as hemolysin/cytolysin (VCC), zonula occludens toxin (Zot), and accessory cholera enterotoxin (Ace) have also been identified. Although cytopathic effects of these toxins have been well characterized *in vitro*, their role regarding the molecular mechanisms underlying the disease pathogenesis is not clear, and requires further research in animal models [5].

Pore-forming toxins (PFTs) are the most common class of toxins that are implicated in bacterial virulence [6]. VCC is an 80 kDa PFT that is expressed in most *V. cholerae* strains including O1 biotype El Tor, O139, and non-O1/non-O139 isolates. VCC peptides, encoded by the *hlyA* gene, assemble into heptameric channels following proteolytic activation by exogenous proteases [7], [8], [9]. The effects of VCC on eukaryotic hosts have been documented at both the cellular and organism level. *In vitro*, VCC is associated with cellular degenerative events such as autophagy, vacuolization, lysis, apoptosis, and necrosis [10], [11], [12], [13], [14], [15]. In infant mouse and rabbit ileal loop models, VCC was found to be responsible for the residual toxicity and diarrhea observed after the administration of vaccine strains into the gastrointestinal system [10].

Additionally, VCC seemed to be the major contributor to the lethality of streptomycin-fed adult mice after gastrointestinal exposure to high doses of El Tor strains [16].

The nematode *Caenorhabditis elegans* has been used as an invertebrate host to identify and assess virulence factors of several human pathogens including *Pseudomonas aeruginosa, Salmonella* Typhimurium *and Yersinia pseudotuberculosis* [17], [18], [19], [20], [21], [22], [23]. *V. cholerae* causes lethal infection in *C. elegans* via a CT and TCP independent process that provides an excellent model to investigate the roles of other *V. cholerae* virulence factors [23]. In this model, the nematode killing by *V. cholerae* has been associated with LuxO regulated genes in the quorum sensing (QS) pathway such as the transcriptional regulator *hapR* and *hapR-*regulated metalloprotease PrtV [23].

We used the CT negative vaccine strains CVD109 and CVD110 to examine the roles of additional *V. cholerae* virulence factors. CVD110 is derived from its parental strain CVD109 with an additional mutation in the *hlyA* locus [24]. In the *C. elegans* infection model, we observed a decrease in nematode killing after feeding CVD110, in comparison to CVD109, pointing to the deleterious effects of *hlyA*. This finding led to the identification of *V. cholerae* hemolysin/cytolysin as a virulence factor that contributes to the pathogenesis of *C. elegans* infection. Using the high throughput Complex Object Parametric Analyzer and Sorter (COPAS) assay and microscopy, we also found that the *hlyA* gene causes growth retardation in *C. elegans*. We determined the severity of lethal infection after feeding nematodes with wild type *V. cholerae* biotypes that have differences in *hlyA* gene structure and expression, and found a correlation between worm lethality and varying levels of bacterial hemolytic activity. Furthermore, we showed that the *hlyA* gene-encoded hemolysin/cytolysin is responsible for the formation of intestinal vacuoles in *C. elegans* during *V. cholerae* infection. Altogether, our findings provide an *in vivo* model for further research on the virulence mechanisms of *V. cholerae* hemolysin/VCC using *C. elegans* as a host organism.

## Materials And Methods

### Bacterial strains, plasmids, media and culture conditions

The bacterial strains and plasmids used in this study are listed in Table 1. *V. cholerae* strains were cultured in tryptic soy broth (TSB, Becton Dickinson Microbiology Systems, BBL, Cockeysville, MD) media supplemented with 1% NaCl at 30°C. *Escherichia coli* OP50 was grown in LB culture medium.

**Table 1:** *C. elegans* and bacterial strains, and plasmids used in this study.

	Relevant genotype and/or phenotype	Source or reference
***C. elegans*** ** strains**		
N2	Wild type Bristol isolate	*Caenorhabditis* Genetics Center
SS104	*glp-4 (bn2)*	*Caenorhabditis* Genetics Center
**Bacterial strains**		
OP50	*E. coli*	*Caenorhabditis* Genetics Center
A1552	*V. cholerae* Wild-type O1 El Tor, Ogawa	Fitnat Yildiz, UCSC
N16961	*V. cholerae* Wild-type O1 El Tor, Ogawa	DVA* Strain collection
569B	*V. cholerae* Wild-type O1 classical	DVA strain collection
VC395	*V. cholerae* Wild-type O1 classical	DVA strain collection
E7946	*V. cholerae* Wild-type O1 El Tor, Ogawa	DVA strain collection
CVD110	Δ*(ctxAB zot ace) hlyA::*(*ctxB mer*) *Hgr* Parental strain: E7946	James B. Kaper, University of Maryland, School of Medicine
CVD109	Δ(*ctxAB zot ace*) Parental strain: E7946	James B. Kaper, University of Maryland, School of Medicine
HNC44	CVD110/pHNC44	This study
HNC45	E7946 Δ*hlyA*	This study
LS38	*S. aureus*	DVA Strain collection
**Plasmids**		
pHNC44	pMMB66EH: *hlyA*	This study
pCWΔ*hlyA*	Δ*hlyA*	K. Satchel, University of Illinois

*Division of Virulence Assessment, CFSAN, FDA.

### Cloning and complementation of *V. cholerae hlyA* gene

For complementation of the *hlyA* mutation in CVD110, a plasmid containing the entire *hlyA* gene was introduced into CVD110. For this procedure, a 2497 bp DNA segment containing the entire *hlyA* gene was amplified from E7946 by PCR using primers VchlySalIF 5′CAGTGTCGACTGACGAGGGTAACCCATGA and VchlyPstIR 5′CAGTCTGCAGTTTCAGGGCATGCTTCCA which were designed to contain SalI and PstI sites (underlined) for subsequent cloning. PCR was performed in 20 µl solutions containing 1 µl of bacterial cell lysate, primers (400 nM) and 17 µl (0.019 U/ml) Platinum Blue PCR Supermix (Invitrogen). PCR conditions for amplification were the following: 95°C for 5 minutes followed by 35 cycles of denaturation at 94°C for 30 seconds; annealing at 56°C for 30 seconds; and extension at 72°C for 60 seconds; with a final extension of 72°C for 7 minutes. The amplified product was cloned into a SalI, PstI digested pMMB66EH [25]. For complementation experiments the resulting plasmid pHNC44 was introduced into a spontaneous streptomycin resistant mutant of CVD110 (resulting in strain HNC44) using a conjugation method described by Datta et al. [26].

### Construction of Δ*hlyA* of *V. cholerae* E7946

HNC45, the strain containing a deletion in *hlyA* locus, was generated via introduction of pCW*ΔhlyA* [16] into a spontaneous streptomycin resistant mutant of E7946 using conjugation. Resulting mutants were analyzed by PCR to ensure the loss of the *hlyA* gene.

### *C. elegans* strains and maintenance

*C. elegans* wild type strain N2 Bristol and SS104 *glp-4(bn4)* strain were obtained from the *Caenorhabditis* Genetic Center (CGC, Minneapolis, MN), and were maintained at 20°C and 16°C, respectively in *C. elegans* habitation media (CeHM) [27] in tissue culture flasks on a platform shaker.

### *Caenorhabditis elegans* Lethality Assay

100 µl of overnight cultures of *V. cholerae* strains were seeded onto the center of the surface of a five cm NGM agar plates and incubated at room temperature (∼22°C) overnight prior to addition of about 50 L4 stage *glp-4(bn2)* worms onto the plates for each treatment (20 to 30 worms per plate). *glp-4(bn2)* temperature sensitive sterile mutants were used to prevent the worms from having progeny during the assay. For lethality assays L4 stage worms were shifted from 16°C to 25°C as soon as they were placed onto the plates. At least three replicates were made for each experimental condition. *E. coli* OP50 (*C. elegans* food strain) was used as a baseline control in each experiment. Plates were incubated at 25°C during the experiment and scored for live worms every 24–48 hours. We censored the missing worms from the analysis at the time of the event. 100 µg/ml ampicillin was added to NGM agar plates used for complementation experiments to maintain the plasmid during the survival assay. The Prism version 4.0 (GraphPad, San Diego, CA) was used to analyze and to plot the data according to a Kaplan-Meier method and survival curves were compared using the logrank test. Statistical significance was set at p-value <0.05.

### Microscopy

Live nematodes were mounted on an agar pad on a slide and covered with a cover glass. Sodium azide was used to anesthetize the worms [28]. L1 stage worms were exposed to test bacteria on NGM agar plates for 48 hours at 20°C before examination. The different developmental stages of *C. elegans* are defined as L1, L2, L3, L4 and adult, and are described as follows [29]. L1 stage: Gonad consists of 4 to 12 cells. L2 stage: More than 12 cells present in the gonad, and vulva development has not started yet. L3 stage: Vulva development is in progress, gonad arms grow towards anterior and posterior ends of the worm. L4 stage: The vulva cells move together and create a channel like opening between uterine cavity and outside environment, gonad arms reflects to grow back towards the middle of the animal where vulva is located, and the somatic gonad is differentiated into the uterus, spermatica and oviduct. Adult stage: Gonad development is complete, oocytes and fertilized eggs are present. Intestinal tracts, somatic gonad and vulva were examined under Nomarski optics using a Zeiss AxioImager D1 microscope (Carl Zeiss MicroImaging, Inc, Thornwood, NY).

### COPAS analysis

A COPAS biosorter [30] (Union Biometrica, MA) was used to assess worm growth. L1 stage animals were synchronized by treating gravid adults with hypochlorite and incubating released eggs in M9 buffer overnight [28]. Experiments were started with synchronized L1 stage animals. After exposure to test bacteria on the NGM agar plates for 72 hours, worms were washed out of the plates into 15 ml Falcon tubes with M9 buffer, washed twice in M9 buffer and sorted following calibration and sample analysis methods [31]. For each experimental condition, worm growth was assessed by measuring the parameter “Extinction” (EXT; represents the optical density of the worms, which measures the decrease in laser light intensity when an object passes through the laser beam). To evaluate the life stage composition of a worm culture, EXT values were plotted against the frequency of events by binning the readings in increments of 50. Sums of EXT values of 1000 worms for each condition were calculated as an index of population growth, and Student's *t* test was used to compare growth.

### Hemolysin assay

CAMP test was performed using *Staphylococcus aureus* strain LS38 on five percent sheep RBC plates according to the procedure described by Christie et al [32]. Staphylococcus aureus strain LS38 was streaked in the middle of the agar plate in a straight line. The *V. cholerae* strain to be tested was streaked perpendicularly to the LS38 streak without allowing the two streaks to touch each other. The presence of enhanced hemolytic reaction was evaluated where the tested bacterial strains are in close proximity with *Staphylococcus aureus* strain LS38, which appears like a clear arrowhead.

## Results

### *hlyA* is required for lethality during *V. cholerae* infection in *C. elegans*

**Figure 1:**
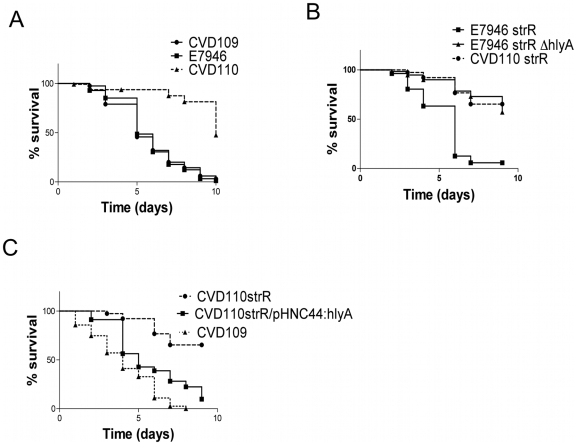
*hlyA* is required for killing during *V. cholerae* infection in *C. elegans*. Lethality analysis was performed in *glp-4(bn2)* worms that were fed with indicated bacterial strains (Table 1). Agar plates were kept at 25°C and scored for survivors at 24–48 hour intervals. Data were plotted according to a Kaplan-Meier method and survival curves were compared using the logrank test. *p*<0.005. CVD110 and CVD109 represent the *hlyA* deficient and *hlyA* containing vaccine strains respectively, and they are isogenic with E7946. strR, streptomycin resistance; Δ*hlyA, hlyA* deletion; CVD110 strR/pHNC44:*hlyA* is the complementation strain. A) Comparison of lethality caused by vaccine strains CVD110 (*hlyA*-), CVD109 (*hlyA*+) and *V. cholerae* WT strain E7946. CVD110 exposed worms: median survival–10 days, CVD109 exposed worms: median survival–5 days, E7946 exposed worms: median survival-5 days. p<0.0001 for CVD110 versus CVD109 and for CVD110 versus E7946. p = 0.3455 for E7946 versus CVD109. B) Comparison of lethality caused by *hlyA* deletion mutant, WT strain E7946 and CVD110. p<0.0001 for E7946 strR versus E7946 strR *ΔhlyA* and for E7946 strR versus CVD110 strR. p = 0.1383 for E7946 strR Δ*hlyA* versus CVD110 strR. C) Comparison of lethality caused by CVD110, CVD110 with *hlyA* expressing plasmid and CVD109. p<0.0001 for each curve comparison in this graph.

Worms fed with a wild type *V. cholerae* strain die faster than the ones fed with *E. coli* OP50, the standard nematode food, or UV-killed *V. cholerae*, and this lethality seems to be the result of a lethal infection that is independent of the major virulence factors, CT and TCP ([23] and our unpublished results). To determine the role of other virulence factors in nematode killing, we fed worms with *V. cholerae* vaccine strains that are deficient in several known virulence factors, and assayed lethality under these conditions. The vaccine strain CVD110 lacks the virulence genes *zot*, *ace, ctxA,* and *hlyA* [24], Table 1. The *ctxB* gene locus was deleted in CVD110 genome, but it was reinserted into the *hlyA* locus to inactivate *hlyA* gene and keep the immunogenicity elicited by CtxB protein. For full toxicity, CtxB requires the presence of CtxA, and since CVD110 does not have *ctxA* gene, the virulence mediated by cholera toxin is lacking in this strain [24]. We observed an attenuated killing response in CVD110 fed worms in comparison to the worms fed with the isogenic wild type *V. cholerae* strain E7946 (Fig. 1A), suggesting that one or more of these deficient factors might be responsible for increased lethality in *C. elegans*. When we fed worms with CVD110's immediate parental strain CVD109, which has an intact hlyA, we found that the presence of hlyA gene was sufficient to kill the worms at a rate comparable to that of observed for *V. cholerae* E7946 (Fig. 1A). To further evaluate the role of *hlyA* gene, we created the E7946-derived strain HNC45 that has a single locus deletion of *hlyA* gene. HNC45 fed worms showed decreased lethality similar to that of observed for CVD110 (Fig. 1B). When we reintroduced a functional copy of *hlyA* into CVD110 via conjugation of a plasmid expressing *hlyA*, the resulting strain showed lethality when fed to *C. elegans* (Fig. 1C) similar to that of the wild type strain E7946. Together, these findings indicated that *V. cholerae hlyA* is responsible for lethality during infection in *C. elegans.*

### *V. cholerae* infection causes developmental delay in *C. elegans* via *hlyA*

**Figure 2:**
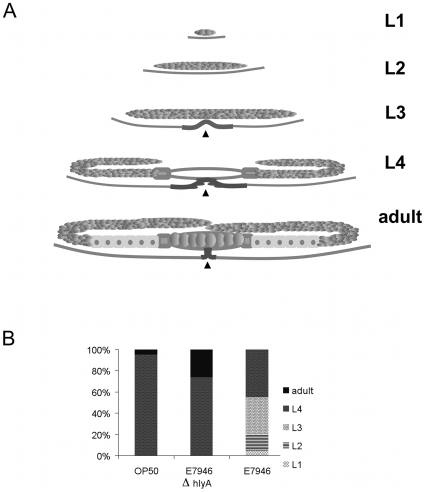
*V. cholerae* causes developmental delay in *C. elegans* via *hlyA* gene. A) Illustrative pictures of gonad and vulva in each developmental stage. Black arrowhead indicates vulva. B) Synchronized L1 stage N2 worms (n = 50 per condition) were fed the indicated bacterial strains on agar plates for 48 hours at 20°C. The developmental stage of growing worms was determined under Nomarski optics using gonad development as milestones.

Because the worm lethality assay measures the longevity of non-reproducing worm populations and allows observations only for adult animals, we wanted to evaluate other possible outcomes in relation to *C. elegans* infection that could be attributed to *hlyA*. To characterize how the exposure to *V. cholerae* affects the life cycle of *C. elegans*, we fed strain E7946 to wild type growing worms and examined their development using two approaches. In the first assay, synchronized L1 stage worms were initiated on bacterial feeding, and were examined under Nomarski optics at 48 hours for subsequent attainment of larval stages. To identify developmental stages, we used the size and shape of the somatic gonad and vulva as developmental landmarks (Methods, and Fig. 2A). While all the worms that were fed with the control bacterium OP50 reached L4 stage and beyond at 48 hours, only 45% of the E7946 fed worms attained L4 stage during this time (Fig. 2B), suggesting that a developmental delay was induced by *V. cholerae* exposure. Using this assay, we evaluated the effects of *hlyA* on larval development and found that all L1 worms that were fed on the *hlyA*-deficient *V. cholerae* strain HNC45 reached L4 stage and beyond at 48 hours (Fig. 2B).

**Figure 3:**
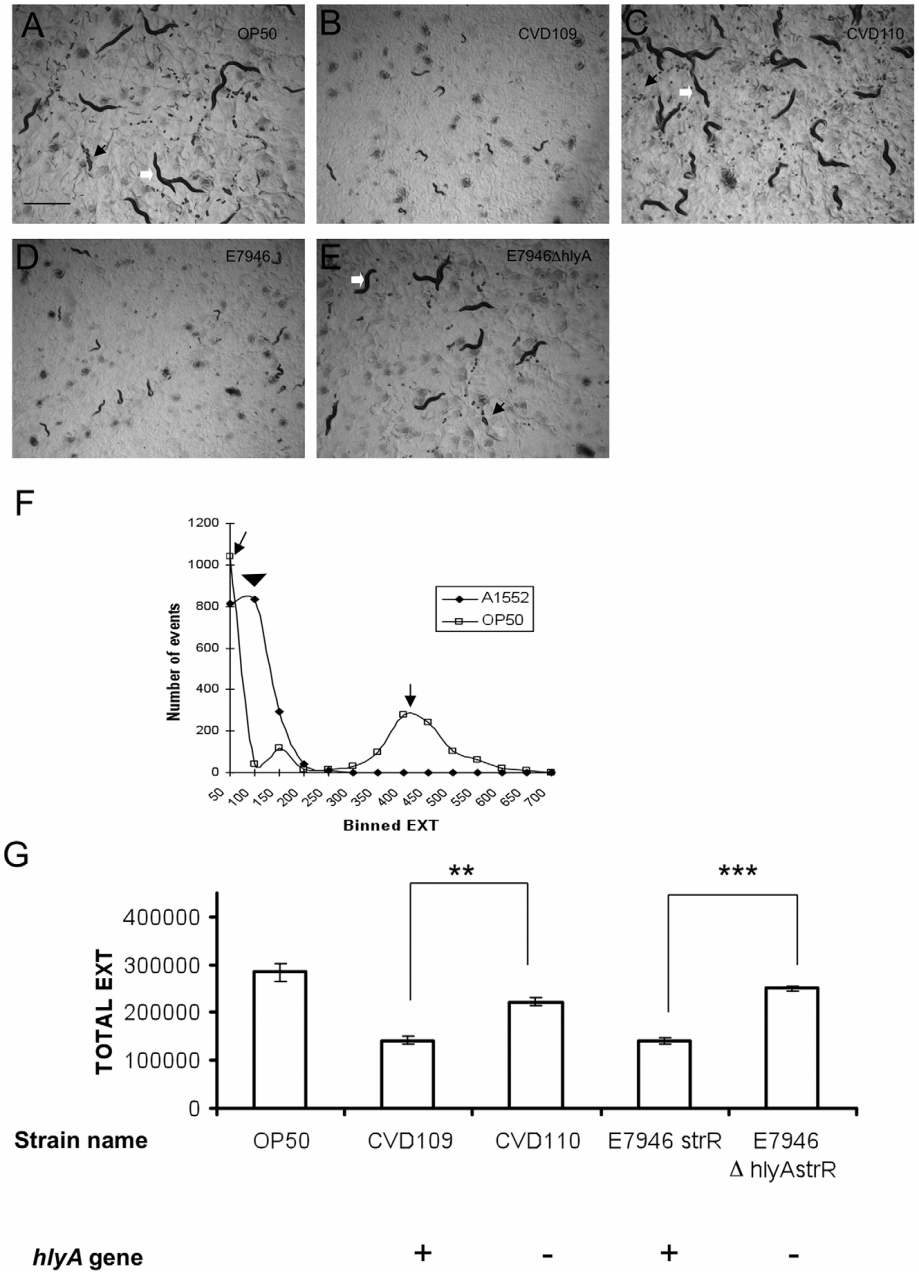
*V. cholerae* causes growth retardation of worm cultures via *hlyA* gene. Synchronized L1 stage N2 worms were fed on indicated bacterial strains on agar plates for 72 hours at 22°C. Worms washed into M9 buffer were sorted using COPAS (n = 1000). Images in A to E indicate the composition of worm cultures prior to sorting. Black arrows indicate eggs, white arrows indicate adult nematodes. (F) Curve representing the optical density (EXT) distribution of sorted worms was plotted. *E. coli* OP50 fed worm populations showed two separable peak domains (indicated with arrows), *V. cholerae* A1552 fed worms showed a single peak (indicated with an arrowhead) falling between *E. coli* OP50 induced peaks that is consistent with a population of worms mostly larger than eggs but fail to reach adult sizes EXT, extinction; strR, streptomycin resistance. (G) Population growth of *C. elegans* fed with *hlyA* deleted, *hlyA* intact *V. cholerae* strains and OP50. Student's *t* test was used to compare growth. ** denotes a statistical significance of P<0.001 according to Student's t test; *** P<0.0001. Total EXT represents the sum of EXT values for sorted worms per condition.

In an independent approach, the COPAS biosorter [30] was used to quantify the optical density distributions of growing worm populations to evaluate the composition of life stages in a given feeding condition. For this assay, synchronized L1 stage worms were fed on *hlyA* intact (E7946 and CVD109) and *hlyA* deficient (CVD110 and HNC45) strains of *V. cholerae* for 72 hours, and the optical density of the worms were evaluated by EXT measurements (Methods). A qualitative assessment of images taken on samples of worm cultures prior to sorting showed that the worms fed with *hlyA* deficient strains and with *E. coli* OP50 contained mostly adults and eggs. In contrast, worms fed with the *hlyA* intact strains contained smaller animals and no eggs (Fig. 3A–E). We observed distinct density distribution curves for *E. coli* OP50 and *V. cholerae* A1552 fed worms, which is consistent with the life stage composition of their respective worm cultures (Figure 3F). We compared the total optical density values that reflects the population growth (methods), and found that the worms exposed to *hlyA(+)* strains showed developmental delay while the worms exposed to *hlyA(-)* strains grew similar to the worms fed with the OP50 bacteria (Fig. 3G). Altogether, these results confirmed our findings in lethality assays, and further implicated a role for *hlyA* as a virulence factor that impairs nematode development.

### Varying levels of hemolytic activity in *V. cholerae* strains correlate with severity of the nematode infection

**Figure 4:**
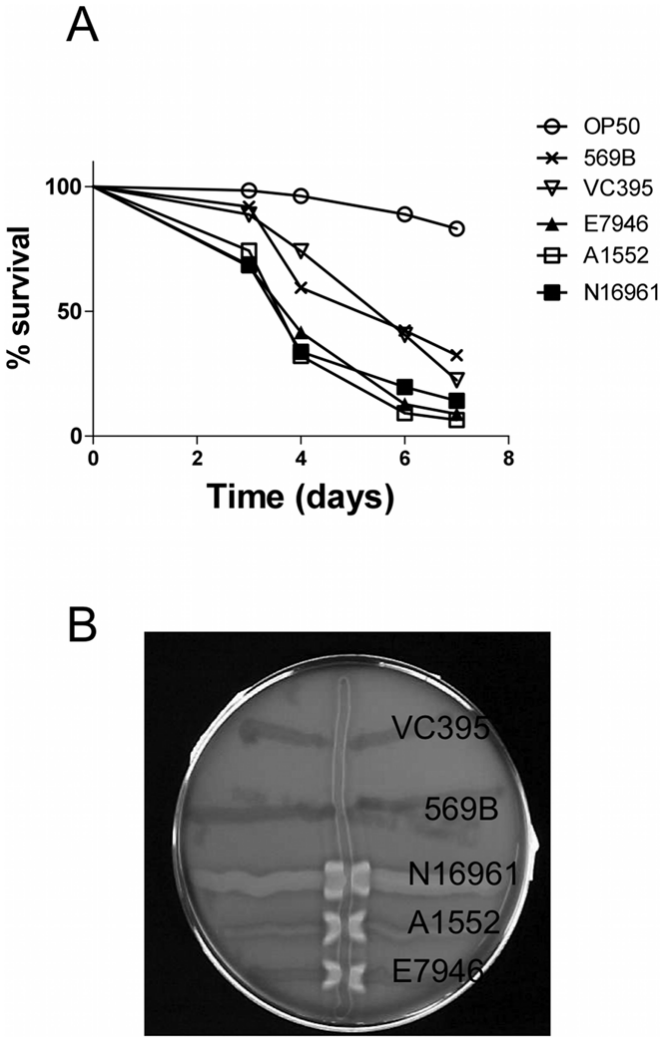
Correlation between level of hemolytic activity and severity of lethality. A) Lethality analysis as described in the legend of Fig. 1 was performed in *glp-4(bn2)* worms that were fed indicated bacterial strains (Table 1). O1 El Tor strains A1552, E7946 and N16961 induce higher lethality than O1 Classical strains 569B and VC395 with a statistical significance of p<0.01 according to logrank test. Curve comparisons within the classical (p = 0.5389) and El tor (p = 0.7224) strains were not statistically significant. B) Hemolytic activity of indicated bacterial strains was determined by the CAMP test (Methods).

One of the major differences between the two biotypes of *V. cholerae* is that the Classical strains exhibit lower hemolytic activity than the El Tor strains [33]. A variation in *hlyA* expression presumably explains the decrease in hemolytic activity, because the levels of *hlyA* expression were found to be lower in Classical biotype than in El Tor biotype [34]. The classical strain 569B also contains a 11 bp deletion in the open reading frame of *hlyA* gene that results in a stop codon and a predicted 244 aa long truncated gene product [35]. Truncated HlyA peptide still has cytotoxic effects, but the molecular lesion lessens the severity of its cytotoxicity [10]. To compare the lethality caused by two biotypes of *V. cholerae*, Classical strains 569B and 395, and El Tor strains N16961, E7946, A1552 were fed to the worms. All the strains tested showed increased lethality in comparison to *E. coli* OP50 baseline, but the Classical strains caused a lower level of lethality than the El Tor strains (Fig. 4A). The results of the CAMP hemolytic assay revealed that while all the El Tor strains had identifiable hemolytic activity, the Classical strains showed no hemolytic activity under assay conditions (Methods; Fig. 4B). Assessing the modulation of the severity of infection by varying levels of hemolytic activity in wild type isolates of *V. cholerae* provided a further clue on how the activity of *hlyA* gene might be related to virulence mechanisms in the nematode infection. Altogether, our results derived from the interactions of different *V. cholerae* isolates with the nematode supported the notion that *hlyA* is a virulence factor in *C. elegans* infection.

### *hlyA* expression contributes to formation of vacuoles in the intestine of *C. elegans*

**Figure 5:**
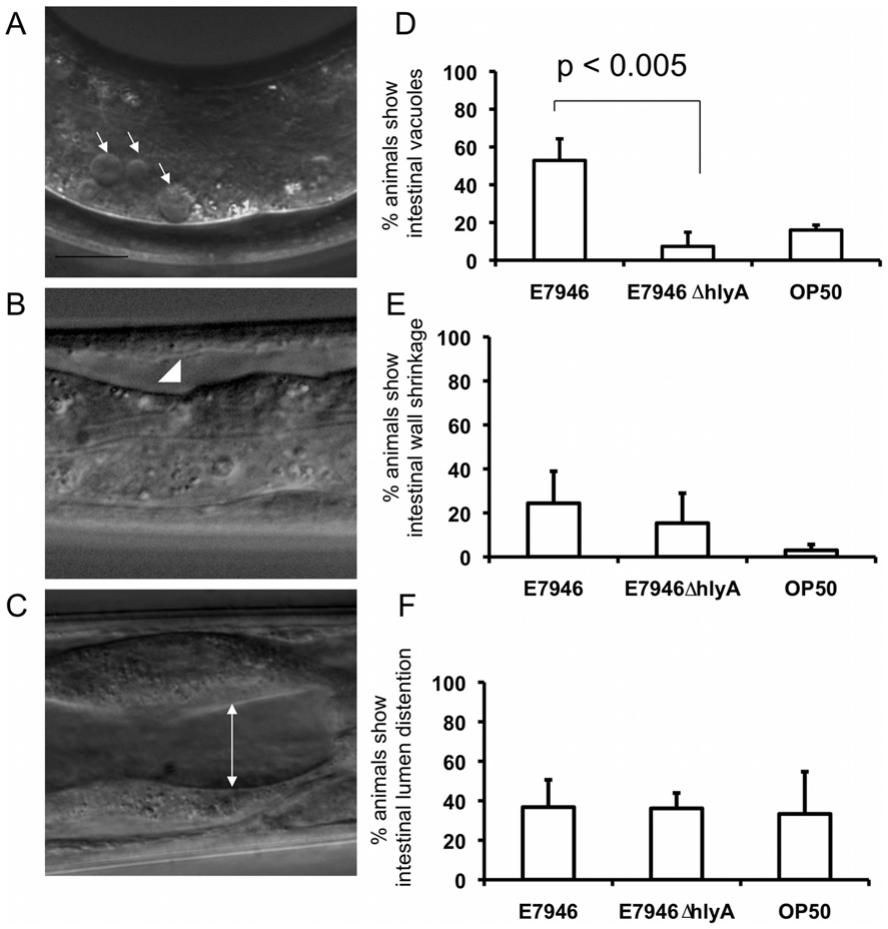
*hlyA* expression is required for formation of vacuoles in the intestine. *glp-4(bn2)* worms were fed wild type strain E7946 and *hlyA* deficient HNC45 for 48 hours at 20°C and examined under Nomarski optics. A) Intestinal vacuoles (as indicated by arrows) appear in the gut of *V. chlorea* fed nematodes. B) Arrowhead marks a region of intestinal wall shrinkage, and C) the extent of a distended lumen is indicated by the white line. Quantifications of these anatomical changes as represented by the percent of animals carrying these changes are shown in D) for intestinal vacuoles, in E) for intestinal wall shrinkage, and in F) for distended lumen. Statistical significance is derived according to Student's t test. n = 20.

*V. cholerae* O1 El Tor strains cause lethality in *C. elegans* through intestinal colonization [23]. In addition, we observed tissue damage in the form of vacuole formation and intestinal wall shrinkage along the gut in worms feeding on wild type *V. cholerae* (Fig. 5A and 5B). Since it has been shown that VCC causes cellular vacuolation in cultured cells [12], [13], [36], we investigated the contribution of *hlyA* to intestinal lesions in *C. elegans*. L1 stage worms were fed with bacterial strains for 48 hours, and examined under Nomarski optics for the presence or absence of anatomical changes that indicate intestinal pathology including appearance of vacuoles, wall shrinkage, and lumen distention. We found that animals fed with the *hlyA* deficient strain HNC45 showed a lower degree of intestinal vacuolization in comparison to the *hlyA* intact E7946-fed worms (Fig. 5A and D), suggesting that the *hlyA* expression may contribute to the formation of intestinal vacuoles during *V. cholerae* infection. There were no statistically significant differences in other anatomical features such as intestinal wall shrinkage, and luminal distention between E7946 and HNC45 fed animals (Fig. 5B, C, E, and F). Together, these results indicate that *hlyA* has a specific role in eliciting intestinal vacuolation during *V. cholerae* infection in *C. elegans* that may represent a crucial step in pathogenesis leading to developmental delay and lethality.

## Discussion

CT is a powerful toxin, and the severity of symptoms induced by it in human gastroenteritis limits our understanding of the role of accessory toxins in the disease process. Although several vaccine studies have uncovered possible roles for accessory toxins as virulence determinants in human diarrheal disease [4], [37], [38], [39], the contribution of these toxins to the pathogenesis of gastroenteritis is not well understood. Since the interaction of *V. cholerae* with various host organisms mimics only partial aspects of the clinical picture in humans, the development of additional host models to examine the mechanisms of virulence is essential. The exposition of the accessory toxin VCC as a virulence determinant via vaccine studies [37] was confirmed by findings in animal and cell culture models [7], [10], [16] and further elucidation of its role in the pathogenesis of *V. cholerae* infection requires the advance of novel animal models. Here, using a CT independent infection model in the nematode *C. elegans*, we demonstrated the *hlyA* gene as a virulence factor contributing to the pathogenesis of infection and lethality in this system. Our findings open the way to further research not only on the interaction of VCC with other proteins on the pathogen side, but also on its interactions and effects regarding host's innate immune system, developmental pathways, and target cells where main events leading to pathogenesis take place.

Previous studies have shown that hemolysins and other members of PFTs produced by several bacterial species induce lethality in *C. elegans* [18], [40], [41], [42]. The *α-hemolysin* of *S. aureus* and the hemolysin ShlA of *S. marcescens* are required for *C. elegans* killing [18], [40], [42]. In addition, *B. thuringiensis* Crystal (Cry) PFTs are toxic to *C. elegans*, producing vacuole-like structures, pitting, and constrictions in the intestinal tissue of the exposed worms [41]. Similarly, we observed tissue damage in the form of vacuoles and constrictions along the intestines of the nematodes that were feeding on wild type *V. cholerae,* and found that this vacuolization was modulated by *hlyA* expression. Our findings are consistent with the results of previous reports stating that VCC cause vacuolization in cultured cells [12], [13], [36], and extend those observations to an *in vivo* experimental setting.

Although the lethality assay is a widely used method in analyzing the host response of *C. elegans* to microorganisms, it measures the longevity of worm populations only in the adult stage. We extended our observations on the *V. cholerae* infection of adult worms to the larval stages of *C. elegans* to assess development. Using microscopy and COPAS to assess population characteristics of growing worms, we showed that *V. cholerae* infection impairs nematode development via *hlyA* gene. Further research is required to investigate whether developmental pathways are altered via VCC during *V. cholerae* infection in *C. elegans*.

What other genes in *V. cholerae* might be interacting with *hlyA* gene during pathogenesis of *C. elegans* infection? A candidate interactor would be PrtV, a protease regulated by the *LuxO–HapR* pathway in *V. cholera* that has been reported to be involved in *C. elegans* lethality [23]. PrtV and VCC may interact by an activating relationship because they both induce lethality in *C. elegans*. A *prtV* mutation in O1 El tor strain O17 does not seem to affect the hemolytic activity of this strain [43], but Ou et al. recently suggested that an 80 kDa protoxin form of VCC be a potential substrate for PrtV using a biochemical assay [44]. Further research is needed to address the interaction of VCC and PrtV *in vivo*.

What happens to cells during PFT exposure? Early effect of pore formation is increased permeabilization of the plasma membrane to ions, in particular calcium, possibly leading to osmotic stress [45]. However, recent studies revealed that cells might be actively responding to the attack of PFTs via major signaling pathways to alleviate cellular destruction [46], [47]. p38 mitogen activated protein kinase (p38 MAPK) pathway has been thought to be a major player in innate immunity, and it was found to be upregulated transcriptionally in response to *B. thuringiensis* Cry5B toxin in *C. elegans* [47]. The same pathway was also shown to be involved in protection of mammalian cells against PFT aerolysin [47]. Cells might deal with PFTs using pathways that regulate cell death. Caspase-1, a major player in apoptosis, appeared to promote cell survival by activating sterol regulatory element binding proteins (SREBPs) upon PFT exposure [46]. Our recent work revealed that hypoxic response pathway protects *C. elegans* against PFTs, including VCC [48]. Cultured cells were found to have an increased autophagic response to VCC, and it was thought that this mechanism was required to override cytotoxicity and prevent cell death [14], [49]. Besides enabling high throughput approaches and powerful genetic and genomic methods, further investigations in *C. elegans -V. cholerae* host pathogen model will be important not only in identifying host immune responses against VCC attack, but also in characterizing the changes underlying pathogenesis at subcellular resolution in a readily accessible way.
